# BLAST Ring Image Generator (BRIG): simple prokaryote genome comparisons

**DOI:** 10.1186/1471-2164-12-402

**Published:** 2011-08-08

**Authors:** Nabil-Fareed Alikhan, Nicola K Petty, Nouri L Ben Zakour, Scott A Beatson

**Affiliations:** 1 Australian Infectious Diseases Research Centre, School of Chemistry and Molecular Biosciences, The University of Queensland, Brisbane, QLD 4072, Australia

## Abstract

**Background:**

Visualisation of genome comparisons is invaluable for helping to determine genotypic differences between closely related prokaryotes. New visualisation and abstraction methods are required in order to improve the validation, interpretation and communication of genome sequence information; especially with the increasing amount of data arising from next-generation sequencing projects. Visualising a prokaryote genome as a circular image has become a powerful means of displaying informative comparisons of one genome to a number of others. Several programs, imaging libraries and internet resources already exist for this purpose, however, most are either limited in the number of comparisons they can show, are unable to adequately utilise draft genome sequence data, or require a knowledge of command-line scripting for implementation. Currently, there is no freely available desktop application that enables users to rapidly visualise comparisons between hundreds of draft or complete genomes in a single image.

**Results:**

BLAST Ring Image Generator (BRIG) can generate images that show multiple prokaryote genome comparisons, without an arbitrary limit on the number of genomes compared. The output image shows similarity between a central reference sequence and other sequences as a set of concentric rings, where BLAST matches are coloured on a sliding scale indicating a defined percentage identity. Images can also include draft genome assembly information to show read coverage, assembly breakpoints and collapsed repeats. In addition, BRIG supports the mapping of unassembled sequencing reads against one or more central reference sequences. Many types of custom data and annotations can be shown using BRIG, making it a versatile approach for visualising a range of genomic comparison data. BRIG is readily accessible to any user, as it assumes no specialist computational knowledge and will perform all required file parsing and BLAST comparisons automatically.

**Conclusions:**

There is a clear need for a user-friendly program that can produce genome comparisons for a large number of prokaryote genomes with an emphasis on rapidly utilising unfinished or unassembled genome data. Here we present BRIG, a cross-platform application that enables the interactive generation of comparative genomic images via a simple graphical-user interface. BRIG is freely available for all operating systems at http://sourceforge.net/projects/brig/.

## Background

With the dramatic improvement of next-generation sequencing technologies over the last five years, there has been a corresponding increase in the amount of publicly available genomic data. As of February 2011, Entrez Genome Projects [1] catalogued 6,071 bacterial and archaeal genome projects. Of these, 1,444 had complete genome sequences, 42 percent of which were released within the last three years. In addition, 3,872 on-going genome projects were registered with the database; 1,734 of which had a draft sequence publicly available. These projects do not include the ten terabase-pairs of sequence data across more than 6,500 entries currently available in the Short Read Archive, the public repository specifically for raw data from next-generation sequencing [2]. Current genome visualisation and data analysis methods are struggling to keep up as it becomes a routine requirement for biologists to compare a new genome to scores, if not hundreds, of other genomes at once.

Genome visualisation methods use linear or circular representations. Linear representations, like those that can be generated using Artemis Comparison Tool (ACT) [3], Genome2D [4], Combo [5], VISTA [6], Mauve [7], BugView [8] and Genomorama [9], have advantages in showing insertions and deletions between genomic sequences and certain programs, like Mauve and ACT, can show genome rearrangements. However, it is difficult to summarise large datasets using these tools. Programs that generate circular figures, like Microbial Genome Viewer [10] and Genome Projector [11], are designed to annotate a single chromosome and have no support for whole genome comparative data. These programs are restricted to published genomes and do not let users analyse their own genomic sequences. DNAPlotter [12] allows the user to input their own genome sequences and can show genome comparisons, but only by generating this information separately and loading it in as custom annotation tracks.

There are comparative circular genome visualisation alternatives available online, such as CGView Server [13] and GeneWiz browser [14], which allow users to upload their own sequences and provide a similar service, although GeneWiz browser can display mapped read data, whereas CGView Server cannot. However, both of these tools are only available as internet resources and limit the number of genome comparisons that can be shown on a single image. Command-line based alternatives and imaging libraries also exist, which require users to prepare all data and customisation through text files, such as Circos [15], CGView [16], Genome Diagram [17] and BLASTAtlas [18]. While these programs are very powerful, they require command-line manipulation and scripting to use, putting them out of reach of many biologist end-users.

To address these issues, we present the BLAST Ring Image Generator (BRIG); an easy-to-use, cross-platform desktop application that enables rapid visualisation of BLAST comparisons to one or more central reference sequences using complete, draft or unassembled genome data.

### Implementation

**Figure 1:**
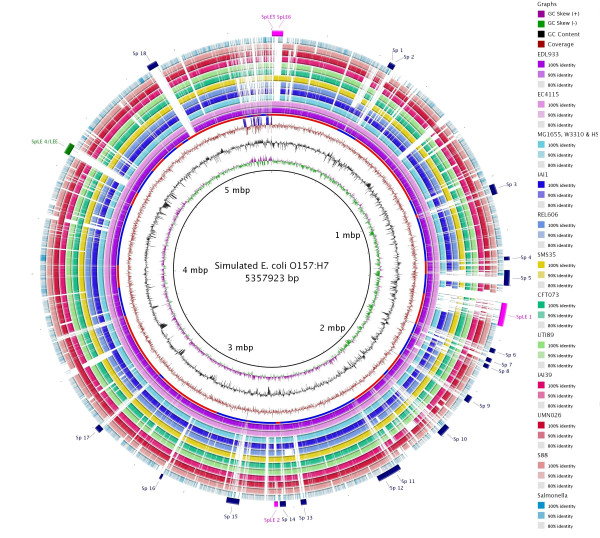
**BRIG output image of a simulated draft *E. coli *O157:H7 str. Sakai genome**. Figure 1 shows a draft *E. coli *genome compared against 27 other prokaryote genomes (the full list of genomes is described in Table 1). The reference genome is an ordered set of contigs, assembled using GS De Novo Assembler (454 Life Sciences/Roche) version 2.3, from simulated sequencing reads generated by MetaSim [21] based on the *E. coli *O157:H7 str. Sakai genome [GenBank:BA000007]. After assembly contigs were ordered against the complete *E. coli *O157:H7 Sakai genome using Mauve [7]. The innermost rings show GC skew (purple/green) and GC content (black). The third innermost ring shows genome coverage (brown); genome regions with coverage more than one standard deviation (~ 41) from the mean coverage (~ 94) are represented as blue spikes. Contig boundaries are shown outside this ring as alternating red and blue bars. The remaining rings show BLAST comparisons of 27 other complete *E. coli *and *Salmonella *genomes against the simulated draft genome assembly (in several cases, multiple genome comparisons are collapsed into a single ring, Table 1). The outermost ring highlights the Sakai prophage, and prophage-like (Sp & SpLE) regions as described by Hayashi *et al*. [20], shown in navy blue and fuchsia, respectively. SpLE 4, containing the locus of enterocyte effacement, is shown in green.

The BLAST Ring Image Generator (BRIG) is a cross-platform desktop application written in Java 1.6. It uses CGView [16] for image rendering and BLAST [19] for genome comparisons. It has a graphical user interface, programmed on the Swing framework, which takes the user step-by-step through the generation of a circular image. The settings used to generate a particular image can be saved for re-use with different genome data, or the entire session can be bundled and saved for later. The image can be generated in JPEG, PNG, SVG or SVGZ format. An example of BRIG's output can be seen in Figure 1. A user guide describing step-by-step tutorials for several visualisation tasks and accompanying example files are provided at http://sourceforge.net/projects/brig/files/.

## Results

### Whole genome comparisons

BRIG is capable of generating circular comparison images for prokaryote genomes, showing multiple genome comparisons in a single image, and displaying similarity between a reference genome in the centre against other query sequences as a set of concentric rings coloured according to BLAST identity. An example image (Figure 1) produced by BRIG shows a comparison of a draft *Escherichia coli *genome with 13 other *E. coli *and 14 *Salmonella *genomes (Table 1). The varying colour gradient of rings 5-16 in Figure 1 indicates a BLAST match of a particular percentage identity, as shown in the key. BLAST matches can be filtered according to a minimum percentage identity or E-value cut-off (or indeed any available BLAST option). These matches are calculated from the perspective of the reference sequence; consequently, regions that are absent from the reference genome but present in one or more of the query sequences will not be displayed. Data from different genomes can be collated into a single lane, which enables visualisation of a large number of genomes and allows users to compare genomes as a group against the central reference sequence. This is shown in Figure 1 where the comparison results from 3 *E. coli *strains, MG1655, HS and W3310, have been grouped together to represent regions of the reference genome that are found in non-pathogenic *E. coli*.

**Table 1:** Genome sequences included in Figure 1

**Ring number**^**1**^	Genome	Accession	Reference
Ring 5	Enterohaemorrhagic *E. coli *O157:H7 str. EDL933	AE005174	[25]

Ring 6	Enterohaemorrhagic *E. coli *O157:H7 str. EC4115	CP001164	J. Craig Venter Institute

Ring 7	Non-pathogenic *E. coli *str. K12 substr. MG1655	U00096	[26]
	
	Non-pathogenic *E. coli *str. K12 substr. W3110	AP009048	[27]
	
	Non-pathogenic *E. coli *HS	CP000802	[28]

Ring 8	Non-pathogenic *E. coli *IAI1	CU928160	[29]

Ring 9	Non-pathogenic *E. coli *B str. REL606	CP000819	[30]

Ring 10	Environmental *E. coli *SMS-3-5	CP000970	[31]

Ring 11	Uropathogenic *E. coli *CFT073	AE014075	[32]

Ring 12	Uropathogenic *E. coli *UTI89	CP000243	[33]

Ring 13	Extraintestinal *E. coli *IAI39	CU928164	[29]

Ring 14	Extraintestinal *E. coli *UMN026	CU928163	[29]

Ring 15	Extraintestinal *E. coli *S88	CU928161	[29]

Ring 16	*Salmonella enterica *subsp. enterica serovar Heidelberg str. SL476	CP001120	J. Craig Venter Institute
	
	*Salmonella enterica *subsp. enterica serovar Dublin str. CT_02021853	CP001144	J. Craig Venter Institute
	
	*Salmonella enterica *subsp. enterica serovar Gallinarum str. 287/91	AM933173	[34]
	
	*Salmonella enterica *subsp. enterica serovar Paratyphi B str. SPB7	CP000886	W.U. Genome Sequencing Center
	
	*Salmonella enterica *subsp. enterica serovar Enteritidis str. P125109	AM933172	[34]
	
	*Salmonella enterica *subsp. enterica serovar cholerasuis str. SC-B67	AE017220	[35]
	
	*Salmonella enterica *subsp. enterica serovar Newport str. SL254	CP001113	J. Craig Venter Institute
	
	*Salmonella enterica *subsp. enterica serovar Typhi str. CT18	AL513382	[36]
	
	*Salmonella enterica *subsp. enterica serovar Typhi str. Ty2	FN424405	[37]
	
	*Salmonella enterica *subsp. enterica serovar Agona str. SL483	CP001138	J. Craig Venter Institute
	
	*Salmonella enterica *subsp. enterica serovar Paratyphi A str. ATCC 9150	CP000026	[38]
	
	*Salmonella enterica *subsp. enterica serovar Typhimurium str. LT2	AE006468	[39]
	
	*Salmonella enterica *subsp. enterica serovar Paratyphi C str. RKS4594	CP000857	[40]

^1 ^Genomes are listed as they appear on Figure 1, from innermost to outermost. Rings 1 to 4 correspond to GC-skew, GC-content, coverage and contig boundaries, respectively.

Users can highlight regions of the reference genome with custom annotations by specifying the label text, colour, shape, and position of features either manually, or by uploading this information as a tab-delimited file. Alternatively, selected annotations can be uploaded from a GenBank or EMBL file; for instance, the annotations shown in the outermost ring in Figure 1 have been read from the GenBank file of *E. coli *O157:H7 str. Sakai [20] by selecting 'misc_features' that contain the text 'Sp' or 'SpLE', which correspond to annotated prophage regions [20].

Generating comparisons of a large number of genomes raises the issue of memory usage. To produce Figure 1, with its comparison against 27 genomes each of approximately 5 Megabase-pairs in size, one Gigabyte of RAM was required on a standard desktop computer. The memory requirement can be reduced by filtering the BLAST results according to E-value and percentage identity cut-offs within BRIG. Alternatively, the amount of memory allocated to BRIG can be altered from within the program.

### A high level of customisation through a user-friendly graphical user interface

A variety of genomic data sources can be used to produce an image, including BLAST comparisons of protein or nucleotide sequences from GenBank, EMBL and FASTA files. BRIG will internally handle all genome comparisons by converting GenBank or EMBL files into FASTA format, creating any necessary BLAST databases, running BLAST and converting the results into a format that CGView renders as the circular image. Users do not have to interact directly with BLAST or CGView, and nor is any knowledge of using command-line programs assumed. By default the central reference sequence is treated as the subject BLAST database with the rings representing matches to individual query sequences.

**Figure 2:**
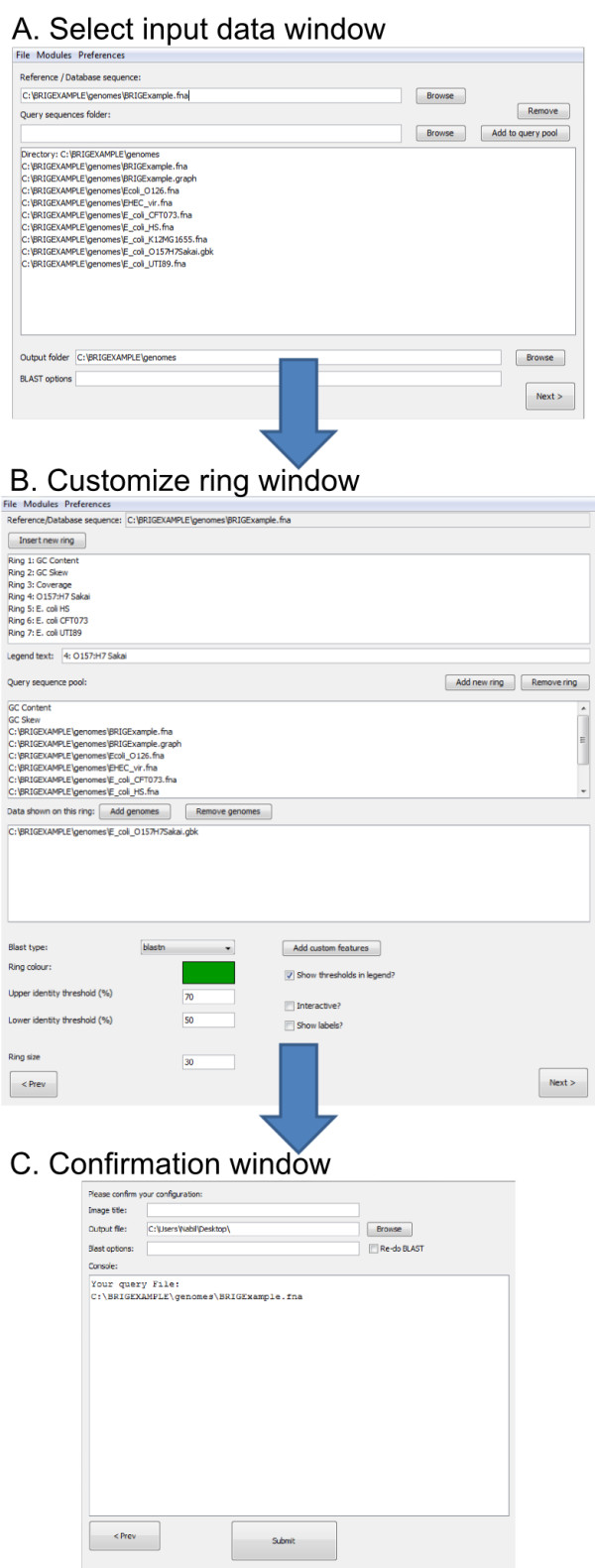
**Screenshots of BRIG's graphical user interfaces**. Screenshots of BRIG's three main graphical user interfaces: A. The "select input data" window where users are able to specify the reference sequences, query sequences, and output folder. B. The "customise ring" window where one or more query sequence files, that were loaded in the previous window, are chosen for each concentric ring. Image drawing configurations, including ring colour, size, identity thresholds and legend text can also be specified. Custom annotations, graphs, or a ring showing contig boundary information can be added at this point. C. In the "confirmation" window settings are confirmed and submitted to BRIG to perform the genome comparisons and image rendering. Progress is written to a console box. From any window prior to job submission, configurations for BLAST or CGview can be altered via the preferences pull-down menu.

Users are taken step-by-step through the process to create a circular comparison image via a graphical user interface (Figure 2). In the first screen (Figure 2A), users specify data they would like to compare to a central reference sequence. In the second screen (Figure 2B), users are able to configure the individual concentric rings; choosing which data they would like to show in each lane and make aesthetic choices including colour or ring size. Lastly, the settings can be reviewed and submitted for BLAST [19] alignment and image drawing using CGView [16] (Figure 2C). Image rendering settings and genome comparison configurations for a particular BRIG image can be saved and reused as an XML profile file. Alternatively, a number of sample templates are available to users, in order to quickly generate an image with optimised size and colour settings.

Users can add their own annotations to a BRIG image through the 'add custom features' dialog, to produce complex yet informative images. Users can alter every aspect of visualisation, including: image size, label visibility, texts and fonts, colours of ring lanes, the gradient reflecting percentage identity, and custom labels inside or outside of a ring.

Data such as transcriptome and microarray expression values can be graphed and displayed as a ring in the circular image. These custom graphs can be produced from user-defined data in a space or tab-delimited file that either includes; the start and stop positions and the value for that region; or a single value for every base pair, with one value per line. To ensure that a useful visualisation is produced regardless of the data source, the default graphing function is to display skew from the mean value, similar to the coverage graph in Figure 1. Users can choose to override this behaviour and scale the graph between zero and a user-defined value.

### Visualisation of information from a user-defined set of sequences

**Figure 3:**
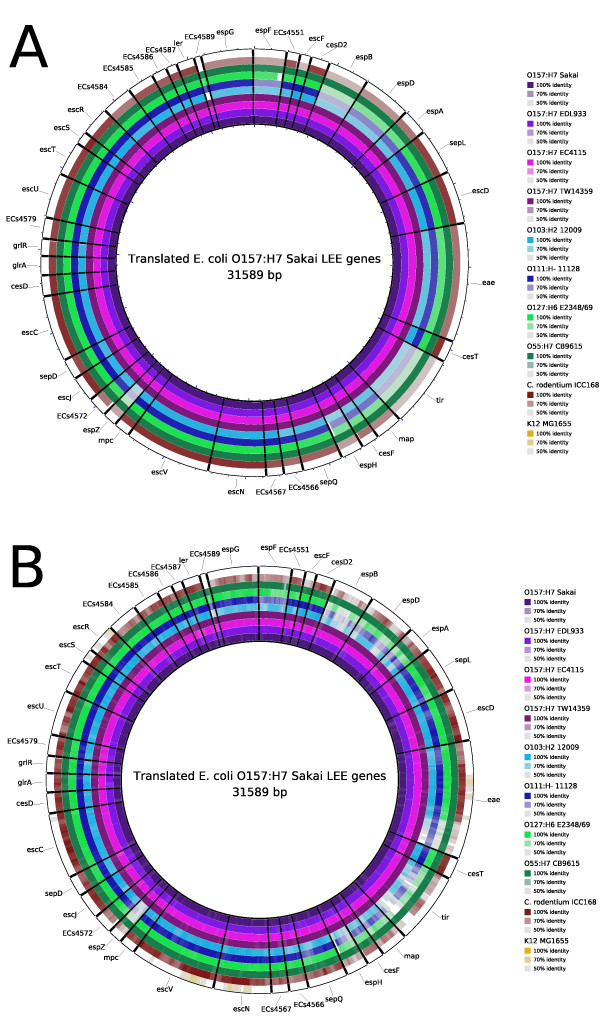
**Using BRIG to compare a multi-sequence reference against complete genomes or unassembled sequence reads**. BRIG image showing the presence, absence and variation of individual genes from the *E. coli *O157:H7 str. Sakai Locus of Enterocyte Effacement (LEE) in related pathogens and *E. coli *K12, a non-pathogenic strain of *E. coli *known to lack the LEE region. Images show a multi-sequence reference consisting of the translated nucleotide sequences of the 41 LEE protein-coding genes, in order, retrieved from the *E. coli *O157:H7 str. Sakai genome [GenBank: BA000007]. Labels around the outside of each circular image correspond to LEE gene names. In both panels the rings display BLAST× comparisons of 10 bacterial genomes with the translated nucleotide sequences of the LEE genes: A. Comparison with complete genome sequences (Table 2). B. Comparison with unassembled, simulated 100 base-pair Illumina reads based on the complete genome sequences used in Figure 3A. The image is scaled to the nucleotide length of the genes. Long tick marks on the outer and inner circumference of the ring indicate increments of 1 kilobase-pairs and short tick marks indicate 200 base-pairs.

In many instances users may only be interested in the presence, absence or variation of a certain set of sequences amongst a number of different genomes. BRIG can visualise this kind of comparison if provided with a multi-FASTA sequence file (of genes, proteins or sequence regions) that will be concatenated to form the central reference ring. An example of such analysis can be seen in Figure 3A, where the translated nucleotide sequences from genes encoded by the Locus of Enterocyte Effacement (LEE) pathogenicity island in the Enterohaemorrhagic *E. coli *strain O157:H7 Sakai genome were compared to the translated nucleotide sequence of whole genomes of other published Enterohaemorrhagic *E. coli*; two Enteropathogenic *E. coli *and one *Citrobacter rodentium *(related bacterial pathogens that also carry the LEE); and *E. coli *K-12 MG1655 (a non-pathogenic strain that does not contain the LEE) Table 2. Comparing translated nucleotide sequences through protein alignment offers better sensitivity for divergent sequences than comparing nucleotide sequences only.

**Table 2:** Table of genomes included in Figure 3 and Figure 4

Ring number	Genome	Accession	Reference
Ring 1	Enterohaemorrhagic *E. coli *O157:H7 str. Sakai	BA000007	[20]

Ring 2	Enterohaemorrhagic *E. coli *O157:H7 str. EDL933	AE005174	[25]

Ring 3	Enterohaemorrhagic *E. coli *O157:H7 str. EC4115	CP001164	J. Craig Venter Institute

Ring 4	Enterohaemorrhagic *E. coli *O157:H7 str. TW14359	CP001368	[41]

Ring 5	Enterohaemorrhagic *E. coli *O103:H2 str. 12009	AP010958	[42]

Ring 6	Enterohaemorrhagic *E. coli *O111:H- str. 11128	AP010960	[42]

Ring 7	Enteropathogenic *E. coli *O127:H6 str. E2348/69	FM180568	[43]

Ring 8	Enteropathogenic *E. coli *O55:H7 str. CB9615	CP001846	[44]

Ring 9	*Citrobacter rodentium *ICC168	FN543502	[45]

Ring 10	Non-pathogenic *E. coli *str. K12 substr. MG1655	U00096	[26]

^1 ^Genomes are listed as they appear on Figures 3 & 4, from innermost to outermost.

BRIG is capable of using raw sequencing reads as query sequences to provide rapid preliminary insights into unassembled draft genome or meta-genome data. To illustrate this feature, we have simulated unassembled Illumina data using MetaSim [21] by randomly sampling one million 100 base pair sequences from the complete genome sequences shown in Figure 3A and applying an Illumina error model. Reads were translated into peptides and used as query sequences in BRIG (using BLASTx) to search against the same central reference sequence in Figure 3A, producing the image shown in Figure 3B. Despite being based only on raw sequencing reads, the representation of sequence presence, absence and variation in Figure 3B is highly similar to that found when using whole genome sequences in Figure 3A.

Figure 3 represents the presence of protein encoding genes within each query genome as a full and vividly coloured bar (e.g. see the *E. coli *O157:H7 strains for the translated *espD *gene). Gene absence can be observed as a blank/white region, like any of the results for *E. coli *K12 MG1665, whose genome does not carry the LEE. Variation in the translated sequences will have a lower sequence identity compared to the reference genome and appear with a fully coloured but slightly faded bar, as seen in Figure 3 for *E. coli *O103:H2 and *C. rodentium *when searching for EspZ, or where the bar is not fully coloured, such as for *E. coli *O111:H- and O127:H6 when searching for EspH. As with any BRIG image, percentage identity cut-off values can be customised to alter the dynamic range of colour shown in each ring. The annotations in Figure 3 illustrate a feature of BRIG where users can opt to load the FASTA headings from a multi-FASTA reference sequence and use these headers to annotate their image.

### Visualisation of information from draft genome assemblies

BRIG is a valuable tool for analysing draft genome sequences. A draft genome that has been assembled into a set of contiguous sequences (contigs) or scaffolds (ordered contigs separated by gaps denoted by N's) in multi-FASTA format can be used as a reference sequence. Contig or scaffold boundaries can be shown as alternating blue or red segments as a custom ring. In addition, by uploading standard genome assembly files (e.g. ACE or SAM), the underlying sequencing reads can be included as a custom graph to show genome coverage. This procedure can help to highlight misassemblies, areas of low coverage and repeat regions that warrant further attention. For instance, the read coverage and contig boundaries in Figure 1 were generated from the ACE file produced by GS De Novo Assembler (454 Life Sciences/Roche). ACE files produced by Consed/Phrap [22] are also acceptable. The reordering of contigs in a draft genome is often carried out after assembly without reordering the corresponding assembly files. To address this, users can use BRIG's graph conversation module to reposition the coverage information from the original ace file to be consistent with the modified draft genome sequence based on a BLASTn comparison.

**Figure 4:**
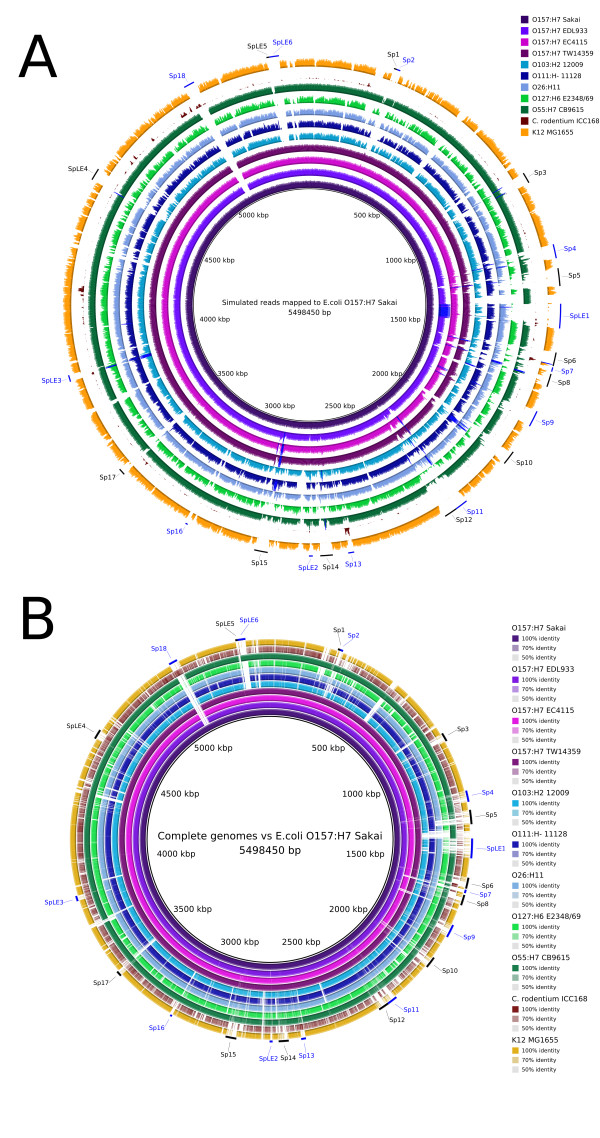
**Using BRIG to map unassembled sequence reads against a complete genome reference**. BRIG images showing genomic regions shared by *E. coli *O157:H7 str. Sakai and related bacteria. The reference sequence is *E. coli *O157:H7 str. Sakai [GenBank: BA000007] with individual rings representing 10 genomes (Table 3). A. Rings show depth of coverage from unassembled, simulated 100 base-pair Illumina reads mapped onto the *E. coli *O157:H7 str. Sakai genome using the BWA [24] read-mapping application. Graph height in each ring is proportional to the number of reads mapping at each nucleotide position in the reference genome from 0 to 30× coverage. Regions with a genome coverage greater than 30× are shown as solid blue bands. B. For comparison, rings show BLASTn comparisons between the same genome sequences used in panel A (Table 3) against the *E. coli *O157:H7 str. Sakai genome. Long tick marks on the outer and inner circumference of the ring indicate increments of 500 kilobase-pairs and short tick marks indicate 100 kilobase-pairs. *E. coli *O157:H7 str. Sakai prophage and prophage-like (Sp & SpLE) regions are annotated in black and blue, respectively, using co-ordinates taken from Hayashi *et al*. [20].

The genome coverage feature of BRIG can also show read mapping information. This can be a useful approach for determining differences amongst multiple unassembled genome datasets relative to the central reference sequence(s). As described previously, BRIG supports read or contig mapping by using BLAST (e.g. Figure 3B). Alternatively, read mapping can be performed externally and read into BRIG as an ACE or SAM file and shown as a coverage graph. ACE files can be produced by the 454 Life Sciences/Roche GS Reference Mapper application, which maps 454 reads to a reference sequence, and there are a number of tools that use the SAM format as the standard file format for mapping short reads to a reference sequence. To illustrate this feature, the simulated reads from Figure 3B were mapped to the *E. coli *O157:H7 Sakai genome [GenBank:BA000007] using BWA and the genome coverage from resulting SAM files was calculated and visualised by BRIG. The resulting image is shown in Figure 4A, where the complete *E. coli *O157:H7 Sakai genome is used as the central reference sequence with the read mapping graphs as rings. These results are broadly comparable to a standard BLAST comparison between O157:H7 Sakai and the original complete genome sequences (Figure 4B). Notably, as a member of a different genus, the genome of *Citrobacter rodentium *is more divergent from the O157:H7 Sakai genome than the other *E. coli *genomes shown and could not be mapped accurately (Figure 4A). This illustrates that the read mapping utility should be restricted to the analysis of strains from the same species, with BLAST being the preferable option for more distant comparisons.

## Discussion

There are already a number of resources that produce circular representations of prokaryote genomes; each with their own unique features and advantages. Table 3 shows a comparison between the major features of BRIG and other GUI or internet based applications that produce circular images for prokaryote genomes. Of these resources, CGView Server [13], GeneWiz Browser [14] and DNAPlotter [12] bear the most resemblance to BRIG.

**Table 3:** GUI and internet-based applications that produce circular comparison images for prokaryote genomes

	BRIG	**DNAPlotter**[12]	**CGView Server**[13]	**GeneWiz Browser**[14]	**Microbial Genome Viewer**[10]	**Genome Projector**[11]
Supports custom annotations	X	X	X	X		X

Search and load annotations from existing files (*e.g*. Genbank, EMBL)	X	X				

Allows users to use their own genome data	X	X	X	X		X

Use Multi-FASTA as reference sequences	X	X				

Internally handles multiple genome comparisons	X		X^1^	X^1^		

Provides percentage identity and e-value filtering for BLAST	X		X	X		

Supports read mapping and visualisation	X			X		X

Natively supports contig and scaffold visualisation	X					

Visualises Clusters of Orthologous Genes (COGS) functional grouping.		X	X	X	X	X

Can produce linear images		X			X	X

Shows an interactive image		X			X	X

^1 ^CGView Server and GeneWiz Browser only support three and seven genome comparisons on a single image, respectively.

BRIG presents a solution to visualising prokaryote genome comparisons for a large number of genomes. Unlike DNAPlotter, BRIG does not show a preview of the image as the user edits it and only produces an image after the user has specified all of their settings. This is a common drawback of other genome comparison applications, including Circos [15], GeneWiz Browser and CGView Server. To address this, image templates are available in BRIG to help first time users to gauge appropriate settings for image aesthetics and scaling. Furthermore, the ability to save template files at any point during a BRIG session enables users to return to previous versions and modify images as needed.

Unlike BRIG, similar tools generally limit the number of genome comparisons that can be shown on a single image and they do not offer the option to collate multiple sequences into a single lane (Table 2). These drawbacks prevent the use of these resources in large-scale genome comparisons that are increasingly necessary as the number of publicly available genome sequences increase. BRIG has been designed with the task of draft genome analysis in mind. GeneWiz Browser, like BRIG, supports mapping and visualising short read sequences onto a reference genome; however, it does not explicitly support easy visualisation of contig boundaries within a reference sequence.

Standard BRIG comparisons rely on BLAST, so an understanding of BLAST parameters and behaviours is required in order to produce informative images. A common pitfall for first time users is the low-complexity filters, which is active by default in BLAST. These filters mask repetitive and low complexity sequences that could cause spurious low-scoring matches when searching large datasets. In BRIG, filtering often results in short (~30 base pairs long) blank regions spanning all query sequences, which may be misinterpreted as unique regions in the reference genome. Filtering can be turned off in the BLAST options field in BRIG. In addition, BLAST comparisons will often produce overlapping hits, which are difficult to visualise on a static flat image. To address this, BRIG was implemented to sort BLAST results so that the highest scoring hits are drawn last by CGView and displayed on top of other lower-scoring matches. As a result, high scoring matches are prominent over low scoring ones.

BRIG is actively maintained with a manual that includes step-by-step tutorials and sample data providing walk-throughs of all the major features. In future we plan to develop support for genome comparisons generated by programs like MUMmer [23] and for BRIG to calculate the co-ordinates of major regions of difference between genomes 'on-the-fly' for use in downstream analyses.

## Conclusions

Here we report the development of the BLAST Ring Image Generator (BRIG), a user-friendly desktop application for comparing and visualising prokaryote genomes using BLAST. BRIG is highly versatile; it can visualise information derived from draft genome data, including contig boundaries, read coverage or read mapping data; it can display the presence, absence or variation of a user-defined set of reference sequences in multiple datasets simultaneously, including unassembled next-generation sequencing reads; and it can display several types of custom graphs and annotations. All facets of the program are customisable through an easy-to-use graphical user interface bringing comparative genome visualisation well within the reach of any user.

## Availability and requirements

**Project name: **BLAST Ring Image Generator (BRIG)

**Project home page: **http://sourceforge.net/projects/brig/

**Operating system(s): **Platform independent

**Programming language: **Java

**Other requirements: **Java 1.6 or greater

**Licence: **GNU GPLv3

**Any restrictions to use by non-academics: **None.

## Authors' contributions

NFA developed and implemented the BRIG application, and helped to draft the manuscript. NKP and NLBZ participated in the design and coordination of the study, and helped to draft the manuscript. SAB conceived the study, participated in its design and coordination, and helped to draft the manuscript. All authors read and approved the final manuscript.
